# Rapid and Cost-Effective *ABO* Blood Genotyping Using a Freeze-Dried, Point-of-Care Ready Loop-Mediated Isothermal Amplification (LAMP) Assay

**DOI:** 10.3390/diagnostics15202568

**Published:** 2025-10-12

**Authors:** Jianlin Zhang, Zhiheng Wang, Yibin Lu, Wei Wu

**Affiliations:** 1 Department of Blood Transfusion, Qingpu Branch, Zhongshan Hospital, Fudan University, Shanghai 201700, China; 2 Clinical Laboratory, Hospital of Obstetrics and Gynecology, Shanghai Medical School, Fudan University, Shanghai 200032, China; 3 Department of Blood Transfusion, Shanghai Ninth People’s Hospital, Shanghai Jiaotong University School of Medicine, Shanghai 200025, China

**Keywords:** loop-mediated isothermal amplification (LAMP), genotyping, point-of-care test (POCT), single nucleotide polymorphism (SNP), freeze-dried reagents

## Abstract

**Background:** The accurate and rapid genotyping of *ABO (chromosome 9q34.2)* blood types is critical for clinical diagnostics and transfusion medicine, particularly in scenarios where serological methods yield uncertain results, such as in neonatal testing or with rare *ABO* subtypes. **Methods:** This study describes a loop-mediated isothermal amplification (LAMP)-based method for *ABO* genotyping that offers a faster and more cost-effective alternative to conventional PCR-based techniques. **Results:** The method targets four key single nucleotide polymorphisms (SNPs) at positions 261, 297, 703, and 930, allowing for the differentiation of common A, B, and O blood types, as well as the rare AB subtype B(A)01. The detection of the B(A)01 subtype is clinically important for preventing transfusion mismatches where serology may be inconclusive. Operating at a constant temperature, the assay can be completed in under an hour without the need for a thermocycler, offering significant time and cost benefits over qPCR. The method demonstrated high specificity, demonstrating detection down to 10 copies across all assays. When validated against a gold-standard method on clinical blood samples, the LAMP assay showed high accuracy (95% C value calculated via binomial exact method): 97.4% for type O, 98.7% for type A, 98.7% for type B, and 100% for the B(A)01 subtype. To enhance usability for point-of-care applications, freeze-dried reagents were developed that permit direct loading of lysed blood samples while maintaining high performance. **Conclusions:** This simplified and robust format positions the LAMP assay as a promising tool for rapid and reliable *ABO* genotyping in diverse clinical settings.

## 1. Introduction

The *ABO* blood group system, identified by Karl Landsteiner in the early 1900s, is the most critical determinant of immunologic compatibility in blood transfusions and organ transplantation [1]. Antigen and antibody-based serological *ABO* blood type detection has been the cornerstone of clinical practices, ensuring safe and adequate blood transfusions by preventing hemolytic reactions and transplant rejections. Biochemically, *ABO* antigens are complex carbohydrates attached to glycolipids and glycoproteins on the surface of various cell types, including RBCs, epithelial cells, and endothelial cells [2]. The expression of these antigens is controlled by the ABO gene, which encodes glycosyltransferase enzymes, such as the A-transferase that adds an N-acetylgalactosamine residue and the B-transferase that adds a D-galactose residue—responsible for the addition of specific sugar residues to the H substance precursor. Variations in the *ABO* gene, including single nucleotide polymorphisms (SNPs), insertions, deletions, and complex allelic combinations, give rise to the diverse A, B, AB, and O phenotypes observed in the population [3]. While serologic testing effectively categorizes individuals within the classical four *ABO* blood groups, it can yield uncertain results in many cases. The limitations of traditional serologic methods are particularly pronounced with the emergence and recognition of numerous *ABO* subgroups [4], which significantly challenge the accuracy of routine blood typing. These subgroups arise from specific variations in the *ABO* gene, which in turn lead to the expression of antigens that may differ from the standard A and B types. Consequently, these subgroups can exhibit varying levels of antigen expression on the surface of red blood cells, a phenomenon that complicates the accuracy of serologic blood typing. When antigen expression is weak or presents as a variant form, standard serological reagents may produce faint, ambiguous, or even false-negative reactions. This complicates interpretation and elevates the risk of mis-typing, thereby undermining a cornerstone of transfusion safety. These challenges are not confined to rare subgroups but are also prevalent in several common clinical scenarios where serology is known to fail or produce uncertain results [5,6]. For example, reliable serological typing is difficult in neonatal patients, individuals who have recently received blood transfusions containing mixed red cell populations, and patients with autoimmune hemolytic anemia where autoantibodies can interfere with testing reagents.

In these complex situations, molecular genotyping of the *ABO* blood group offers a valuable alternative by directly analyzing the genetic determinants underlying *ABO* phenotypes [7,8]. Over the past decades, advancements in molecular diagnostics, including PCR-based techniques and next-generation sequencing (NGS) [9], have facilitated the identification and characterization of numerous *ABO* alleles. While powerful, the integration of these technologies into routine practice has been slowed by significant logistical obstacles. Methods like qPCR and NGS often involve substantial expense, extended processing times, and a reliance on dedicated instrumentation and highly trained personnel. These genetic approaches enable precise differentiation of genotypes such as AA versus AO and BB versus BO, which are indistinguishable through serological methods alone [10]. Although *ABO* genotyping offers promise, its integration into routine healthcare practice has been slowed by logistical obstacles. These include substantial expense, extended processing durations, reliance on dedicated instrumentation and trained staff, a lack of comprehensive regulatory standards, and the nonexistence of uniform competency assessment schemes.

Loop-mediated isothermal amplification (LAMP) emerges as a promising solution to address some challenges of *ABO* genotyping, particularly by offering a faster, cost-effective, and simpler alternative to both serological and polymerase chain reaction (PCR) based molecular techniques [11]. Developed by Notomi et al. [12], LAMP is a nucleic acid amplification method that operates under isothermal conditions (between 60 and 65 °C), eliminating the need for expensive thermocycling equipment required by traditional PCR and quantitative PCR (qPCR) methods. LAMP employs a set of four to six specially designed primers that recognize distinct regions of the target DNA, enhancing both the specificity and efficiency of the amplification process. This method accelerates the amplification process, often completed in under an hour, and simplifies the assay workflow, making it more accessible for rapid and point-of-care applications.

This study addresses a critical gap in blood typing diagnostics: the need for a method that combines the accuracy of molecular genotyping with the speed, low cost, and simplicity required for point-of-care (POC) applications. While serology suffers from inaccuracies in complex cases, advanced molecular techniques like qPCR are often too slow and costly for routine or field use. Therefore, the central hypothesis of this work is that a loop-mediated isothermal amplification (LAMP) assay [13,14], enhanced with a simplified lysis protocol and freeze-dried reagents [15,16], can provide a robust, rapid, and accessible solution for *ABO* genotyping. To test this hypothesis, this study leverages the inherent advantages of LAMP to overcome the limitations of both serological and PCR-based methods. By targeting key SNPs within the *ABO* gene, the developed LAMP assay offers a highly specific and sensitive solution for genotyping, achieving a limit of detection (LoD) of 10 copies. Furthermore, adopting a simplified lysis approach eliminates complex sample preparation steps, and the development of a stable, freeze-dried reagent format enhances usability for resource-limited settings. Our method uniquely integrates direct blood lysis, freeze-dried reagents, and detection of rare B(A) subtypes, together demonstrating the potential for a portable and remotely deployable ABO genotyping system.

## 2. Materials and Methods

### 2.1. Plasmid Cloning

Template plasmids were cloned as templates used in LAMP reactions. The backbone fragment was PCR generated from pUC57 plasmid by using primer ZJL093 and primer ZJL094 (Supplementary Table S1) [17]. The template fragments (261_del_fragment, 261_G_fragment, 297_A_fragment, 297_G_fragment, 703_G_fragment, 703_A_fragment, 930_A_fragment and 930_G_fragment in Supplementary Table S1) were synthesized from Genewiz, Suzhou, China. These 8 template fragments were assembled with the backbone fragment [18], which resulted the 8 template plasmids (pZJL126, pZJL127, pZJL128, pZJL129, pZJL130, pZJL131, pZJL132 and pZJL133). The pZJL126 and pZJL127 plasmids are positive and negative templates for 261 delete reaction, the pZJL128 and pZJL129 plasmids are positive and negative templates for 297 A reaction, the pZJL130 and pZJL131 plasmids are positive and negative templates for 703 G reactions, and the pZJL132 and pZJL133 plasmids are positive and negative templates for 930 A reaction. These 4 plasmids (pZJL126, pZJL128, pZJL130, pZJL132) were also diluted to 5 different concentrations from c1 to c5 (The corresponding copy number is from 10,000 copies to 1 copy) for LoD tests. Plasmid concentrations were initially quantified using a Qubit dsDNA HS Assay Kit (Thermo Fisher Scientific, Waltham, MA, USA) for high-sensitivity fluorometric measurement, followed by the above serial dilutions to achieve the specified copy numbers based on plasmid size.

### 2.2. LAMP Primer Design

For the design of LAMP primers targeting the 4 SNP positions (261, 297, 703, and 930), five primer sets were initially generated using the online tool available at https://lamp.neb.com (accessed on 21 December 2023) (NEB, Ipswich, MA, USA) for each target [19]. These initial primer sets were then subjected to in silico optimization using Oligo 7 software (Molecular Biology Insights, Cascade, CO, USA) to minimize the potential for hairpin formation and to prevent primer-dimer formation [20]. All primers were also BLASTed against human genome (NCBI, *e*-value < 10^−5^) to minimize off-targets. Following initial optimization, the thermodynamic properties of the candidate primer sets were rigorously evaluated using the Multiple Primer Analyzer (Thermo Fisher Scientific, Waltham, MA, USA) to select the single best set for each target SNP [21]. The selection criteria were stringent to ensure high specificity and amplification efficiency. A primer set was only considered favorable if the Gibbs free energy (ΔG) for any significant secondary structure, such as hairpins or dimers, was greater than −5.0 kcal/mol, which minimizes the risk of non-specific amplification. Furthermore, the melting temperatures (Tm) of all primers within a set were required to be within the optimal range of 60–65 °C and not differ by more than 3 °C from one another to ensure uniform annealing. The primer set that best satisfied these specific thermodynamic parameters was selected for experimental validation. To differentiate the deletion at 261, and the difference between G and A at 297, 703 and 930, we used the 1st nucleobase mismatch at the 3′ of FIP to achieve the goal. The final primer information is in Supplementary Table S4 (LAMP primers).

### 2.3. Whole Blood Lysis

Whole blood samples were collected from consenting donors into EDTA-containing tubes and stored at −20 °C. For lysis, 100 µL of preserved or clotted whole blood was mixed with an equal volume of Allele-In-One Human Blood Direct Lysis Buffer from Allele Biotech (San Diego, CA, USA) in a 600 µL microcentrifuge tube [22,23]. The mixture was thoroughly vortexed while adhering to biosafety protocols to avoid aerosol generation and incubated at 55 °C for 15 min to lyse cells and release DNA. Cellular debris was then separated from the DNA by centrifugation at 12,000× *g* for 2 min, resulting in a supernatant containing the released DNA. The clear supernatant was used directly as a template for LAMP reactions, with 2 µL added per reaction. Appropriate personal protective equipment (PPE) was worn throughout, biosafety guidelines were followed, and biological waste was disposed of according to hospital protocols to prevent contamination and health risks.

### 2.4. LAMP Reaction

This study used SYBR Green I for quantitative LAMP reaction with a qPCR machine. A reaction mix is prepared, containing 1× Isothermal Amplification Buffer, 1.4 mM dNTP mix, 0.5 M Betaine, primers at specified concentrations (0.2 µM for F3 and B3, 1.4 µM for FIP and BIP, 0.8 µM for Loop primers), 4 U of Bst 2.0 DNA polymerase (NEB, Ipswich, MA, USA), 1× SYBR Green I, and nuclease-free water to a total volume of 18 µL per reaction [24]. Two microliters of the DNA template (Blood lysis supernatant) are added to each well, making a final reaction volume of 20 µL. The setup is then placed in a qPCR machine set for isothermal amplification at 65 °C for 40 min, with real-time fluorescence readings taken every minute to quantify the amplification. This reaction temperature was selected as it is the manufacturer-recommended optimum for Bst 2.0 DNA Polymerase, ensuring maximal amplification efficiency; this was confirmed in our preliminary experiments. Post-amplification, a 5 min incubation at 95 °C was used to inactivate the reaction samples as a biosafety practice. No-template controls (NTCs) were included in every run to monitor for contamination and non-specific amplification.

### 2.5. LAMP Reagent Freeze-Dry

The freeze-dried LAMP master mix was prepared by first combining the reaction components in a clean environment, including 1× Isothermal Amplification Buffer, 1.4 mM dNTP mix, 0.5 M Betaine, optimized primer concentrations (0.2 µM each for F3 and B3, 1.4 µM each for FIP and BIP, 0.8 µM each for LF and LB), 4 U of Bst 2.0 DNA Polymerase per reaction (NEB, Ipswich, MA, USA), 1× SYBR Green I, and nuclease-free water. Cryoprotectants were then added to the mix, including 5 mM D-(+)-Trehalose dihydrate, 3.2 mM Dextran-40, and 1.2 mM Polyethylene glycol-2000 (PEG-2000), followed by aliquoting into PCR tubes (20 µL each) [25,26]. The tubes containing the LAMP reagents were frozen at −80 °C prior to lyophilization and then subjected to a primary drying phase under vacuum at −60 °C for 16 h, followed by secondary drying at −20 °C for another 6 h. The temperature was then slowly increased (1 °C per minute) to room temperature (25 °C). The PCR tubes with the dried product were capped under vacuum to prevent rehydration and packaged in vacuum-sealed plastic bags. For use, the freeze-dried master mix was rehydrated with molecular-grade water, supplemented with 2 µL of DNA template in 18 µL nuclease-free water, mixed gently, and loaded into the LAMP reaction as described above. The freeze-dried reagents maintained full functionality with no significant loss of performance after storage for 90 days at 4 °C and 7 days at 25 °C, with plans for extended testing. (All cryoprotectants were purchased from Sangon Biotech, Shanghai, China).

### 2.6. Clinical Sample Collection and Ethical Approval

A total of 78 clinical whole blood samples were obtained from the blood bank at the Qingpu Branch of Zhongshan Hospital, Fudan University, for the validation phase of this study. Of the 78 samples, there were 34 type O, 19 type A, 21 type B, and 4 B(A) subtype (confirmed by NGS or allele-specific PCR as gold-standard for rare variants). This study, involving human blood samples, was conducted in strict accordance with the Declaration of Helsinki and received formal approval from the Institutional Review Board (IRB) of the Qingpu Branch, Zhongshan Hospital (IRB approval number: ZSQP_IRB_WZ_20240618). All samples were processed and analyzed following stringent ethical guidelines to ensure participant privacy and data confidentiality.

## 3. Results and Discussions

### 3.1. Detection Strategy for ABO Blood Type SNP Patterns

Sequencing analyses of *ABO* blood types have revealed specific single nucleotide polymorphisms (SNPs) associated with each of the four blood types. For blood type A, the primary SNP identified is rs8176746 (c.796C > A, p.Leu266Met), which modulates glycosyltransferase activity and enables the expression of the A antigen. Blood type B corresponds to rs8176747 (c.803G > C, p.Gly268Ala), which is responsible for B antigen expression. AB blood type, expressing both A and B antigens, harbors both rs8176746 (c.796C > A, p.Leu266Met) and rs8176747 (c.803G > C, p.Gly268Ala) SNPs. Conversely, blood type O is characterized by the rs8176748 (c.261delG) variant, which results in a non-functional glycosyltransferase and the absence of A and B antigens [27,28]. The wealth of existing public sequencing data from the scientific literature and databases has revealed additional SNPs, enabling finer subtyping of *ABO* blood types. For the proof of concept of our LAMP-based *ABO* blood genotyping, Table 1 summarizes four key SNPs (261, 297, 703, and 930) that account for 3 *ABO* blood types and 1 rare blood type. For instance, the deletion at position 261 in Exon 6 is characteristic of blood type O, though subtypes such as O3 retain a G at this position. Additionally, while nucleotide 297 in Exon 6 is consistently A for blood type A, it remains identical between subtypes O1 and O1V. Nucleotides 703 and 930 in Exon 7 are both A in blood type B [29]. The rare B(A)01 subtype, a variant of AB blood type, differs from blood type B by having a G at position 703 in Exon 7. To differentiate these SNPs, we designed a LAMP detection strategy (Table 2). This method employs SNP-specific primers that introduce a deliberate mismatch at the 3′ end of the forward inner primer (FIP), enabling the selective detection of target SNP at a specific position. Positive or negative LAMP reactions at each SNP site are combined to determine the corresponding blood type.

**Figure 1:**
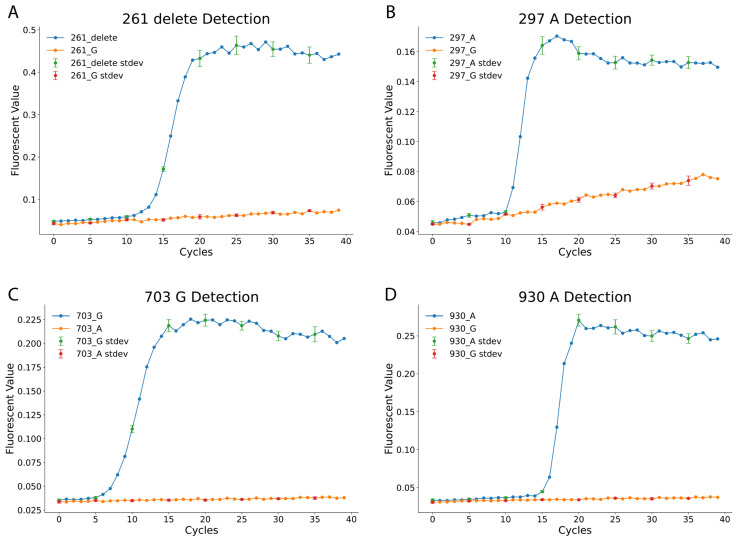
Specificity of Individual LAMP Assays for *ABO* SNP Detection. This figure demonstrates the high specificity of the four loop-mediated isothermal amplification (LAMP) assays designed for key *ABO* single nucleotide polymorphism (SNP) detection. Each panel shows a specific assay tested against its target DNA template versus a non-target template. (**A**) The 261_delete assay shows rapid fluorescence amplification only in the presence of the O-allele template (261_delete), with no signal from the non-target G-allele template (261_G). (**B**) The 297_A assay specifically amplifies the A-allele template (297_A), while the non-target G-allele template (297_G) shows a flat, negative curve. (**C**) The 703_G assay is highly specific for the common G-allele template (703_G), showing no cross-reactivity with the A-allele template (703_A). (**D**) The 930_A assay specifically detects the B-allele marker (930_A) and does not amplify the non-target G-allele template (930_G). Collectively, these results confirm that each LAMP assay is highly specific to its intended SNP target. Data points represent the mean of three replicate experiments, and the standard deviation is shown with error bars every 5 min. No-template controls (NTCs) were included in all experiments and showed no amplification (flat lines).

Figure 1 illustrates the effectiveness of the LAMP assay in distinguishing the 4 SNPs. The 261_delete reaction targets the deletion of G at position 261 in Exon 6, while the 297_A reaction detects an A at position 297 in Exon 6. Similarly, the 703_G and 930_A reactions identify G and A nucleotides at positions 703 and 930 in Exon 7, respectively. Each LAMP reaction was performed under standardized conditions, with fluorescence signals recorded every minute for 40 min using a qPCR machine, corresponding to 40 amplification cycles. The results confirm the specificity of the designed LAMP reactions. For example, in the 261_delete assay, samples with the deletion exhibited strong amplification, while samples with a G at the same position showed no amplification. Similar trends were observed in the 297_A, 703_G, and 930_A assays, with fluorescence curves distinctly separating SNP variants.

### 3.2. Analytical Sensitivity of the Designed LAMP Reactions

**Figure 2:**
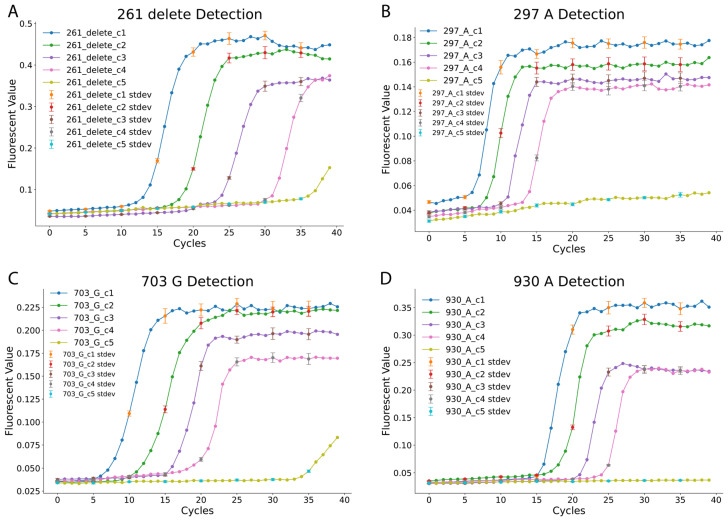
Analytical Sensitivity of Each LAMP SNP Assay. This figure demonstrates the analytical sensitivity of each of the four SNP-specific LAMP assays using a five-point serial dilution of synthetic DNA templates. The concentrations tested were c1 (10,000 copies/reaction), c2 (1000 copies), c3 (100 copies), c4 (10 copies), and c5 (1 copy). (**A**) Analytical sensitivity for the 261_delete assay. (**B**) Analytical sensitivity for the 297_A assay. (**C**) Analytical sensitivity for the 703_G assay. (**D**) Analytical sensitivity for the 930_A assay. All four assays demonstrate a clear dose-dependent response, where higher initial DNA concentrations result in faster amplification (a lower cycle number). Consistent and reliable amplification was observed down to 10 copies per reaction (c4), suggesting high sensitivity for the assays. The 1-copy concentration (c5) showed stochastic, inconsistent amplification. Error bars (every 5 min) indicate the standard deviation of triplicate measurements. No-template controls (NTCs) were included in all experiments and showed no amplification (flat lines).

After verifying the LAMP reactions’ ability to differentiate specific nucleotides at the designated SNP positions, the analytical sensitivity of the four LAMP reactions was evaluated [30]. The positive template plasmids (pZJL126, pZJL128, pZJL130, and pZJL132) representing blood type SNPs were serially diluted to five concentrations, ranging from 1 to 10,000 copies (c1: 10,000 copies; c2: 1000 copies; c3: 100 copies; c4: 10 copies; c5: 1 copy). Each dilution point for the sensitivity analysis was performed in triplicate to ensure statistical validity. The fluorescence amplification curves displayed in Figure 2 indicate a consistent increase in cycle threshold as sample concentration decreases. The reactions exhibited distinct characteristics: the 297_A and 703_G reactions detected targets within 5–10 min at high concentrations, while the 261_delete reaction required approximately 13 min to detect targets at similar concentrations. The 930_A reaction was the slowest, requiring more time for detection across all concentrations. Despite these variations, consistent detection was observed down to 10 copies within the 40 min reaction time cutoff. In contrast, the 1-copy dilution provided inconsistent signals and was therefore deemed below the reliable limit of detection under our assay conditions, reinforcing the robustness of the assay’s established high sensitivity.

Interestingly, for the 261_delete and 703_G reactions, low-level fluorescence signals were observed at the lowest concentration (c5, 1 copy). However, these signals were inconsistent and deemed unreliable under the 40 min cutoff. The results further highlight the reliability and specificity of the LAMP reactions for detecting individual SNPs at varying concentrations. These findings validate the use of this LAMP-based method for precise *ABO* blood type genotyping, even at low DNA input levels, which enabled us to conduct blood sample tests.

### 3.3. Blood Sample Test by Using the LAMP Blood Typing Method

**Figure 3:**
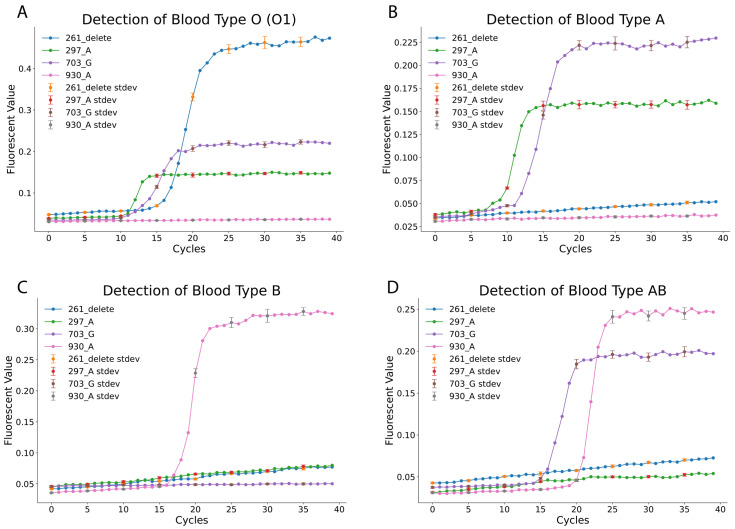
Validation of the LAMP-Based *ABO* Genotyping Panel on Human Blood Samples. This figure demonstrates the successful application of the complete four-assay LAMP panel to determine the *ABO* blood type from genomic DNA extracted from clinical blood samples. Each panel displays the combined fluorescence amplification results from all four SNP assays run on a single sample, with the resulting pattern determining the genotype. (**A**) Detection of Blood Type O (O1): Strong positive signals are observed for the 261_delete, 297_A, and 703_G assays, with the 930_A assay remaining negative, correctly identifying the O allele. No-template controls (NTCs) were included in all experiments and showed no amplification (flat lines). (**B**) Detection of Blood Type A: Positive amplification is seen for the 297_A and 703_G assays, a pattern characteristic of the A allele. (**C**) Detection of Blood Type B: The sample shows a positive signal only for the 930_A assay, with the 261_delete, 297_A, and 703_G assays remaining negative, identifying the B allele. (**D**) Detection of Blood Type AB: The presence of both A and B alleles is confirmed by positive signals in the 703_G and 930_A assays, while the 261_delete and 297_A assays are negative. These distinct amplification patterns validate the assay’s accuracy and utility for *ABO* genotyping in a clinical context. Error bars (every 5 min) represent the standard deviation of triplicate experiments.

To evaluate the practical application of the LAMP-based *ABO* blood typing method, actual blood samples were tested using the developed assay. Compared to qPCR, the LAMP method demonstrated superior resistance to contamination and sample residue interference [11]. A simplified lysis strategy was employed, utilizing the Allele-In-One human blood direct lysis buffer to lyse whole blood samples. The lysed samples were then directly loaded into the designed LAMP reactions for genotyping. The fluorescence amplification curves (Figure 3) clearly differentiated blood types. For blood type O (O1), positive amplification was observed for the 261_delete, 297_A, and 703_G reactions, while the 930_A reaction remained negative (as summarized in Table 1 and Table 2). Similarly, blood type A samples showed positive results for the 297_A and 703_G reactions but tested negative for the 261_delete and 930_A reactions. Blood type B was identified through positive amplification of only the 930_A reaction, with all other reactions negative. The rare AB subtype B(A)01 exhibited a distinct pattern, with positive amplification for the 703_G and 930_A reactions, while the 261_delete and 297_A reactions were negative. These findings confirm the assay’s ability to accurately differentiate blood types based on unique SNP-specific profiles.

Supplementary Table S2 summarizes the method’s accuracy and specificity. Across all tested samples, the LAMP-based method demonstrated exceptional performance, achieving 100% accuracy and specificity for the rare AB subtype B(A)01. Blood types O, A, and B exhibited accuracy rates of 97.4%, 98.7%, and 98.7%, respectively, with corresponding specificity rates of 97.7%, 98.3%, and 98.2%. These results validate the robustness of the LAMP-based approach, which combines high sensitivity and specificity with simplified sample preparation. The simplified lysis strategy eliminates the need for complex workflows, making this method well-suited for routine diagnostic applications and point-of-care settings. This study highlights the potential of the LAMP-based method as a rapid, reliable, and accessible tool for *ABO* blood typing in both clinical and field environments.

### 3.4. Freeze-Dried LAMP Reagents for Simplified ABO Blood Type Detection

**Figure 4:**
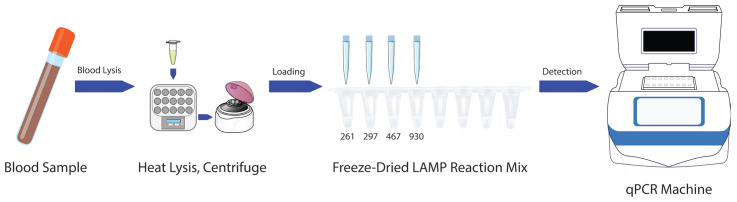
Schematic workflow for ABO blood typing using freeze-dried LAMP reagents. Whole blood samples undergo lysis, followed by heat incubation at 55 °C for 15 min and centrifugation at 12,000× *g* for 2 min to obtain DNA-containing supernatant. The supernatant is then loaded onto pre-aliquoted freeze-dried LAMP reaction mixes targeting SNPs (261, 297, 703, and 930) for isothermal amplification and detection.

To further simplify the LAMP-based *ABO* blood typing method, a freeze-dried reagent format was developed, enabling direct sample loading following blood lysis (Figure 4). The freeze-dried reagents were prepared by incorporating stabilizing reagents, including D-(+)-Trehalose dihydrate (5 mM), Dextran-40 (3.2 mM), and Polyethylene glycol-2000 (1.2 mM), into the LAMP reaction mixture [15]. This approach ensured reagent stability and preserved the functional integrity of the assay under ambient storage conditions. The freeze-dried reagents were evaluated using lysed blood samples prepared with the Allele-In-One human blood direct lysis buffer. The samples were directly added to the pre-aliquoted freeze-dried reaction mixes and reconstituted rapidly upon contact with the liquid sample.

The results, summarized in Supplementary Table S3, demonstrated that the freeze-dried LAMP reagents retained full functionality, achieving high accuracy and specificity across all tested blood types. Blood type O showed an accuracy of 96.2% and a specificity of 95.6%, while blood types A and B achieved accuracy rates of 98.7% and 97.5%, respectively, with corresponding specificities of 98.3% and 96.6%. The AB subtype B(A)01 maintained 100% accuracy and specificity, underscoring the robustness of the freeze-dried format. Although the accuracy and specificity for blood types O and B were slightly lower than the unfrozen LAMP reagents, the differences are reasonable given the effects of stabilizing reagents used in the freeze-drying process. The cryoprotectant cocktail (trehalose, dextran, PEG) used in the lyophilization process may affect the reaction kinetics upon rehydration. The long-term stability of our reagents was tested by comparing the reaction for reagents at 4 °C for 90 days, at 25 °C for 7 days and fresh prepared samples. The results at Supplementary Figure S1 showed that there is minimal difference (McNemar’s test, *p* > 0.05) between the 3 batches of samples with tests with primer sets 261 and 297 (Primer sets information in Supplementary Table S1). These results support that our cryoprotectant cocktail recipe are stable for long term at 4 °C and short term at 25 °C.

This freeze-dried format offers several advantages, including enhanced reagent stability, ease of transport, and reduced dependence on cold chain logistics. The combination of simplified lysis and freeze-dried reagents eliminates the need for complex sample preparation, making the method particularly suitable for point-of-care and field-based diagnostics. By maintaining high accuracy and specificity across both common and rare blood types, the freeze-dried LAMP reagents represent a significant advancement in simplifying *ABO* blood typing and increasing its accessibility. These innovations highlight the potential for wide-scale implementation of the method in diverse clinical and resource-limited settings.

In comparison to existing ABO genotyping methods, standard serological testing offers low cost (~$5–10 per test) and rapid turnaround (10–30 min) using basic lab supplies but can yield inconclusive results in complex cases like neonatal or subgroup typing. In contrast, qPCR provides molecular precision at a higher cost (~$20–50 per test) and longer time (2–4 h) requiring expensive thermocyclers (~$10,000–50,000), while NGS enables comprehensive allele detection but is cost-prohibitive (~$100–500 per test) with extended processing (24–72 h) and sequencer equipment (~$100,000+). Our LAMP assay bridges these gaps with comparable low cost (~$5–15 per test, including freeze-dried reagents), faster completion (<1 h), and minimal equipment needs (basic incubator ~$500–2000), making it particularly suitable for resource-limited settings, though it has lower throughput than NGS; the freeze-dried format further enhances practicality by reducing cold-chain logistics and enabling direct sample loading as described earlier.

## 4. Conclusions

This study establishes a robust and reliable loop-mediated isothermal amplification (LAMP)-based method for *ABO* blood typing by targeting key single nucleotide polymorphisms (SNPs) associated with blood types. The detection strategy, leveraging SNP-specific primers, effectively differentiates common and rare *ABO* subtypes, including the rare AB subtype B(A)01, with high specificity and sensitivity. The method demonstrates detection down to 10 copies, enabling accurate genotyping even with low DNA input levels. Testing on actual blood samples validated the assay’s practical application, demonstrating 100% accuracy for the rare AB subtype and high accuracy rates of 97.4%, 98.7%, and 98.7% for blood types O, A, and B, respectively. These findings confirm the reliability of this method for routine diagnostic applications. Further simplifying the assay, a freeze-dried LAMP reagent format was developed, maintaining full functionality under ambient storage conditions and ensuring high accuracy and specificity for all tested blood types. Although slight reductions in performance were observed for blood types O and B compared to the unfrozen reagents, these differences were minimal and expected due to the stabilizing reagents used in the freeze-drying process. The freeze-dried format offers several operational advantages, including enhanced stability, ease of transport, and reduced dependence on cold chain logistics, making it highly suitable for point-of-care and field-based diagnostics.

Overall, this LAMP-based method provides a rapid, cost-effective, and accessible solution for *ABO* blood typing. Its high accuracy, simplified workflow, and adaptability to resource-limited settings position it as a valuable tool in clinical and transfusion medicine. Our future work will focus on the necessary steps required for clinical validation and broader implementation. Before this assay can be used in a hospital setting, extensive validation is required, which will involve several key steps. This includes further optimization of the freeze-dried reagents and expanding the SNP panel to include additional rare subtypes, such as cis-AB (e.g., via SNPs at 467C > T and 803G > C) or A3 (e.g., 871G > A). Larger-scale validation studies are needed. This will involve a prospective study with a diverse patient cohort, including complex cases such as post-transfusion patients, and direct head-to-head comparisons against gold-standard molecular methods on all samples. While the reagents demonstrated stability for 90 days at 4 °C and 7 days at 25 °C, long-term room temperature stability (e.g., 1–6 months) was not evaluated in this study and represents a limitation. Extended tests of aged or degraded samples are planned for future optimization.

This study represents a pilot-scale validation with a modest sample size (n = 78) from a single-center cohort of East Asian ancestry, which may not capture the full genetic diversity of global populations. Rare subtypes beyond B(A)01 were underrepresented. Future work will involve multi-center trials with ethnically diverse cohorts (e.g., including South Asian, Caucasian, African, and Latin American participants) to confirm robustness and identify potential allele frequency variations that could affect assay performance. For practical point-of-care (POCT) application, a primary objective is the development of a portable fluorescent incubator to replace the reliance on a qPCR machine. We also plan to integrate the method with automated platforms to prepare for large-scale applications and incorporate machine learning algorithms for enhanced data analysis and more accurate blood group typing. These development efforts will be guided by the necessary regulatory pathways for clinical adoption. These rigorous steps are essential for enhancing this diagnostic tool’s scalability and validating its clinical impact, paving the way for its potential adoption in diverse healthcare settings.

## Figures and Tables

**Table 1:** *ABO* Blood Type SNP Pattern.

Blood Type	Nucleotide Position and SNP			
	Exon 6		Exon 7	
	261	297	703	930
O (O1, O1V)	delete	A	G	G
A (A1, A2)	G	A	G	G
B	G	G	A	A
AB [B(A)01]	G	G	G	A

**Table 2:** LAMP *ABO* Blood Type Detection.

Blood Type	LAMP Detection			
	261_Delete	297_A	703_G	930_A
O (O1, O1V)	delete	A	G	G
A (A1, A2)	G	A	G	G
B	G	G	A	A
AB [B(A)01]	G	G	G	A

## Data Availability

Data supporting this study are available from the corresponding author upon request.

## Supplementary Materials

The following supporting information can be downloaded at: https://www.mdpi.com/article/10.3390/diagnostics15202568/s1, Figure S1: Stability tests with 261 and 297 set primers; Table S1: Primers and Fragments for Cloning; Table S2: ABO Blood genotyping data summary; Table S3: ABO Blood genotyping data summary with freeze-dried LAMP mixture; Table S4: LAMP Primers.
