# Supplementary material for: Rapid and Cost-Effective ABO Blood Genotyping Using a Freeze-Dried, Point-of-Care Ready Loop-Mediated Isothermal Amplification (LAMP) Assay
Source: Diagnostics (Basel). 2025 Oct 12;15(20):2568. doi: 10.3390/diagnostics15202568 (PMC12563363; doi:10.3390/diagnostics15202568)
Supplement: Supplementary file 1 [file diagnostics-15-02568-s001.zip › diagnostics-3875198-supplementary.pdf]

Loop-mediated isothermal amplification-based ABO blood genotyping

Jianlin Zhang [1,*], Zhiheng Wang [2], Yibin Lu [3], Wei Wu[1*]

[1] Department of Blood Transfusion, Qingpu Branch, Zhongshan Hospital, Fudan University, Shanghai, China

[2] Clinical laboratory, Hospital of Obstetrics and Gynecology, Shanghai Medical School, Fudan University, Shanghai, China

[3] Department of Blood Transfusion, Shanghai Ninth People's Hospital of Shanghai Jiaotong University School of Medicine, Shanghai, China

* Corresponding Author, e-mail: wuwei2257@hotmail.com, zhangjl1228@hotmail.com

Contributing authors, e-mail: Zhiheng Wang: wzh.0409@163.com

Yibin Lu: luyib@me.com

Table S1. Primers and Fragments for Cloning

Primer or Fragment Name	Sequences
ZJL093	5' CCGCCAACACCCGCTGA 3'
ZJL094	5' GGTGCCTAATGAGTGAGC 3'
261_del_fragment	GCAGACAAGCCCGTCAGGGCGCGTCAGCGGGTGTTGGCGGACCGCAC GCCTCTCTCCATGTGCAGTAGGAAGGATGTCCTCGTGGTACCCCTTGGC TGGCTCCCATTGTCTGGGAGGGCACATTCAACATCGACATCCTCAACGA GCAGTTCAGGCTCCAGAACACCACCATTGGGTTAACTGTGTTTGCCATC AAGAAGTAAGTCAGTGAGGTGGCCGAGGGTAGAGACCCAGGCAGTGG CGAGGTGCCTAATGAGTGAGCTAACTCACATTAATTGCGTTGC
261_G_fragment	GCAGACAAGCCCGTCAGGGCGCGTCAGCGGGTGTTGGCGGACCGCAC GCCTCTCTCCATGTGCAGTAGGAAGGATGTCCTCGTGGTGACCCCTTGG CTGGCTCCCATTGTCTGGGAGGGCACATTCAACATCGACATCCTCAACG AGCAGTTCAGGCTCCAGAACACCACCATTGGGTTAACTGTGTTTGCCAT CAAGAAGTAAGTCAGTGAGGTGGCCGAGGGTAGAGACCCAGGCAGTG GCGAGGTGCCTAATGAGTGAGCTAACTCACATTAATTGCGTTGC
297_A_fragment	GCAGACAAGCCCGTCAGGGCGCGTCAGCGGGTGTTGGCGGTAGGAAG GATGTCCTCGTGGTGACCCCTTGGCTGGCTCCCATTGTCTGGGAGGGCA CATTCAACATCGACATCCTCAACGAGCAGTTCAGGCTCCAGAACACCAC CATTGGGTTAACTGTGTTTGCCATCAAGAAGTAAGTCAGTGAGGTGGCC GAGGGTAGAGACCCAGGCAGTGGCGAGTGACTGTGGACATTGAGGTCT CTCCTTGTGTTCAAGACAGAGTGGGGTGGTGCCTAATGAGTGAGCTAAC TCACATTAATTGCGTTGC
297_G_fragment	GCAGACAAGCCCGTCAGGGCGCGTCAGCGGGTGTTGGCGGTAGGAAG GATGTCCTCGTGGTGACCCCTTGGCTGGCTCCCATTGTCTGGGAGGGCA CGTTCAACATCGACATCCTCAACGAGCAGTTCAGGCTCCAGAACACCAC CATTGGGTTAACTGTGTTTGCCATCAAGAAGTAAGTCAGTGAGGTGGCC GAGGGTAGAGACCCAGGCAGTGGCGAGTGACTGTGGACATTGAGGTCT CTCCTTGTGTTCAAGACAGAGTGGGGTGGTGCCTAATGAGTGAGCTAAC TCACATTAATTGCGTTGC
703_G_fragment	GCAGACAAGCCCGTCAGGGCGCGTCAGCGGGTGTTGGCGGCCACGTG GGCGTGGAGATCCTGACTCCGCTGTTCGGCACCCTGCACCCCGGCTTCT ACGGAAGCAGCCGGGAGGCCTTCACCTACGAGCGCCGGCCCCAGTCCC AGGCCTACATCCCCAAGGACGAGGGCGATTTCTACTACCTGGGGGGGT TCTTCGGGGGGTCGGTGCAAGAGGTGCAGCGGCTCACCAGGGCCTGCC ACCAGGCCATGATGGTCGACCAGGCCAAGGTGCCTAATGAGTGAGCTA ACTCACATTAATTGCGTTGC
703_A_fragment	GCAGACAAGCCCGTCAGGGCGCGTCAGCGGGTGTTGGCGGCCACGTG GGCGTGGAGATCCTGACTCCGCTGTTCGGCACCCTGCACCCCAGCTTCT ACGGAAGCAGCCGGGAGGCCTTCACCTACGAGCGCCGGCCCCAGTCCC AGGCCTACATCCCCAAGGACGAGGGCGATTTCTACTACCTGGGGGGGT TCTTCGGGGGGTCGGTGCAAGAGGTGCAGCGGCTCACCAGGGCCTGCC

	ACCAGGCCATGATGGTCGACCAGGCCAAGGTGCCTAATGAGTGAGCTA ACTCACATTAATTGCGTTGC
930_A_fragment	GCAGACAAGCCCGTCAGGGCGCGTCAGCGGGTGTTGGCGGGCCAACG GCATCGAGGCCGTGTGGCACGACGAGAGCCACCTGAACAAGTACCTGC TGCGCCACAAACCCACCAAGGTGCTCTCCCCCGAGTACTTGTGGGACCA GCAGCTGCTGGGCTGGCCCGCCGTCCTGAGGAAGCTGAGGTTCACTGC GGTGCCCAAGAACCACCAGGCGGTCCGGAACCCGTGAGCGGCTGCCA GGGGCTCTGGGAGGGCTGCCGGCAGCCCCGTCGGTGCCTAATGAGTGA GCTAACTCACATTAATTGCGTTGC
930_G_fragment	GCAGACAAGCCCGTCAGGGCGCGTCAGCGGGTGTTGGCGGGCCAACG GCATCGAGGCCGTGTGGCACGACGAGAGCCACCTGAACAAGTACCTAC TGCGCCACAAACCCACCAAGGTGCTCTCCCCCGAGTACTTGTGGGACCA GCAGCTGCTGGGCTGGCCCGCCGTCCTGAGGAAGCTGAGGTTCACTGC GGTGCCCAAGAACCACCAGGCGGTCCGGAACCCGTGAGCGGCTGCCA GGGGCTCTGGGAGGGCTGCCGGCAGCCCCGTCGGTGCCTAATGAGTGA GCTAACTCACATTAATTGCGTTGC

Table S2. ABO Blood genotyping data summary

Blood types	Number of positive samples	Tested positive	True negative	False positive	False negative	Accuracy	Specificity	Positive predictive value (PPV)	Negative predictive value (NPV)
O (O1, O1V)	34	33	44	1	1	97.5%	97.8%	97.1%	97.8%
A (A1, A2)	19	19	59	1	0	98.7%	98.3%	95.0%	100%
B	21	21	57	1	0	98.7%	98.3%	95.4%	100%
AB [B(A)01]	4	4	74	0	0	100%	100%	100%	100%

Table S3. ABO Blood genotyping data summary with freeze-dried LAMP mixture

Blood types	Number of positive samples	Tested positive	True negative	False positive	False negative	Accuracy	Specificity	Positive predictive value (PPV)	Negative predictive value (NPV)
O (O1, O1V)	34	33	44	2	1	96.2%	95.6%	94.3%	97.8%
A (A1, A2)	19	19	59	1	0	98.7%	98.3%	95.0%	100%
B	21	21	57	2	0	97.5%	96.6%	91.3%	100%
AB [B(A)01]	4	4	74	0	0	100%	100%	100%	100%

Table S4: LAMP Primers

Targets	F3	FIP	LF	B3	BIP	LB
261	CCTCTCTCCATGTGCAGT	CGTTGAGGATGTCGATGTTGAAGAAGGATGTCCTCGTGGTA	ACAATGGGAGCCAGCCAAG	CCACTGCCTGGGTCTCTA	CAGTTCAGGCTCCAGAACACCTCGGCCACCTCACTGACT	GGTTAACTGTGTTTGCCATCAAG
297	GTGGTGACCCCTTGGCTG	CCAATGGTGGTGTTCTGGAGCATTGTCTGGGAGGGCACA	TCGTTGAGGATGTCGATGTTG	TCTGTCTTGAACACAAGGAGAG	TGTTTGCCATCAAGAAGTAAGTCAGATGTCCACAGTCACTCGCC	CGAGGGTAGAGACCCAGGC
703	CGTGGAGATCCTGACTCCG	CGTTGAGGATGTCGATGTTGAAGAAGGATGTCCTCGTGGTA	ACAATGGGAGCCAGCCAAG	CCACTGCCTGGGTCTCTA	CAGTTCAGGCTCCAGAACACCTCGGCCACCTCACTGACT	GGTTAACTGTGTTTGCCATCAAG
930	GGCATCGAGGCCGTGTG	TGCTGGTCCCACAAGTACTCGGCCACCTGAACAAGTACCTA	GCACCTTGGTGGGTTTGTGG	GGGGCTGCCGGCAGCCCTC	CGTCCTGAGGAAGCTGAGGTGAGCCCCTGGCAGCCGCTCA	GGTGCCCAAGAACCACCAG

Figure S1. Stability tests with 261 and 297 set primers

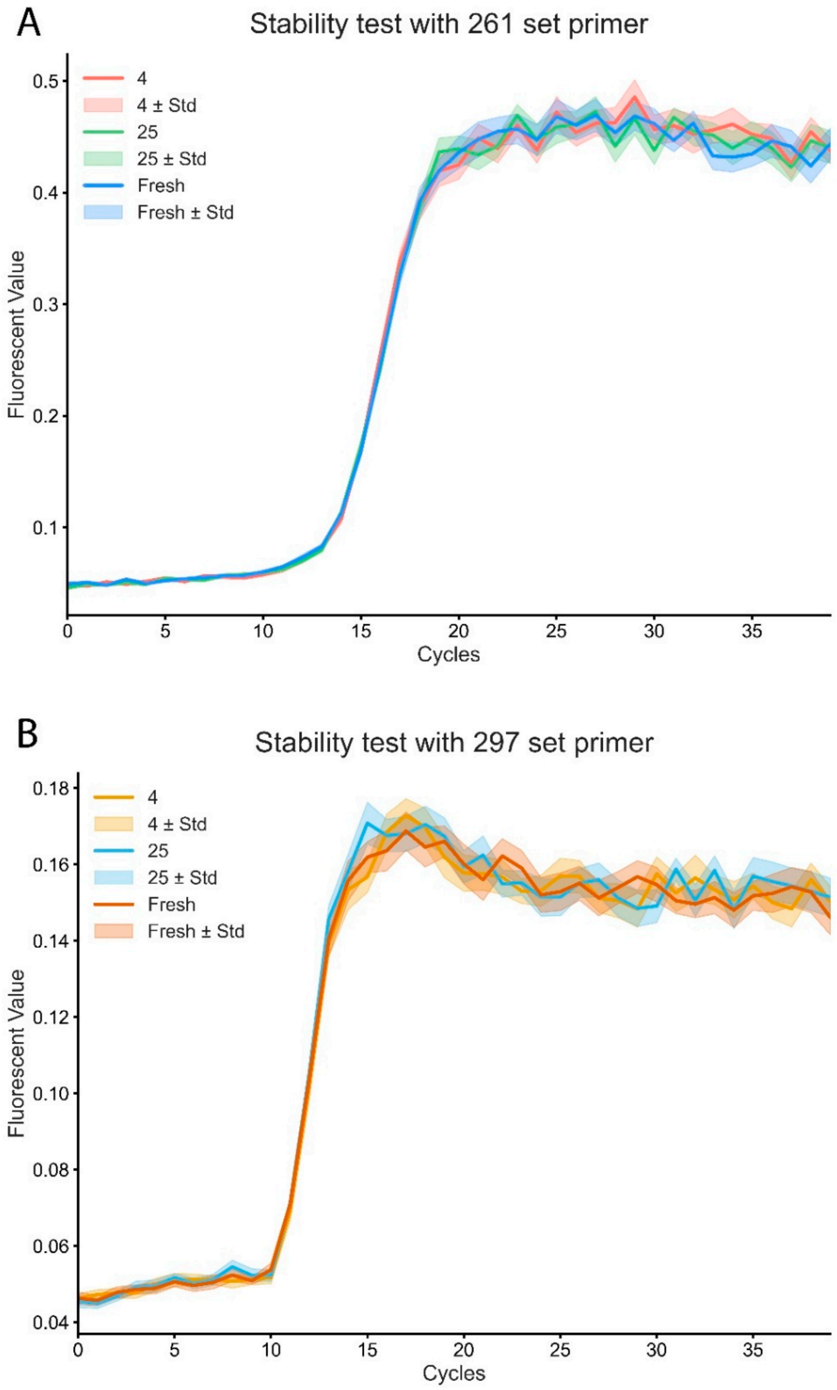

Legend annotations: '4': 4°C for 90 days sample fluorescent value;

'4 ± Std': 4°C for 90 days sample fluorescent value stdev;

'25': 25°C for 7 days sample fluorescent value;

'25 ± Std': 25°C for 7 days sample fluorescent value stdev;

'Fresh': fresh prepared sample fluorescent value;

'Fresh ± Std': fresh prepared sample fluorescent value stdev.

Panel A is data from 261 set primer, Panel B is data from 297 set primer.

No-template controls (NTCs) were included in all experiments and showed no amplification (flat lines).
